# An improved RT-qPCR method for direct quantification of enveloped RNA viruses

**DOI:** 10.1016/j.mex.2022.101737

**Published:** 2022-05-23

**Authors:** Pavlina Gregorova, Minna-Maria K. Heinonen, L. Peter Sarin

**Affiliations:** RNAcious Laboratory, Molecular and Integrative Biosciences Research Programme, Faculty of Biological and Environmental Sciences, University of Helsinki, Finland

**Keywords:** RT-qPCR, Direct quantification, Bacteriophage, Phi6, RNA virus, RNA-dependent RNA polymerase

## Abstract

Reverse transcription quantitative PCR (RT-qPCR) has emerged as the gold standard for virus detection and quantification, being utilized in numerous diagnostic and research applications. However, the direct detection of viruses has so far posed a challenge as the viral genome is often encapsidated by a proteinaceous layer surrounded by a lipid envelope. This necessitates an additional and undesired RNA extraction step prior to RT-qPCR amplification. To circumvent this limitation, we have developed a direct RT-qPCR method for the detection of RNA viruses. In our method, we provide a proof-of-concept using phage phi6, a safe-to-use proxy for pathogenic enveloped RNA viruses that is commonly utilized in e.g. aerosolization studies. First, the phage phi6 envelope is removed by 1% chloroform treatment and the virus is then directly quantified by RT-qPCR. To identify false negative results, firefly luciferase is included as a synthetic external control. Thanks to the duplex format, our direct RT-qPCR method reduces the reagents needed and provides an easy to implement and broadly applicable, fast, and cost-effective tool for the quantitative analysis of enveloped RNA viruses.

• One-step direct RT-qPCR quantification of phage phi6 virus without prior RNA isolation.
• Reduced reaction volume for sustainable and cost-effective analysis.

## Graphical abstract

**Figure:**
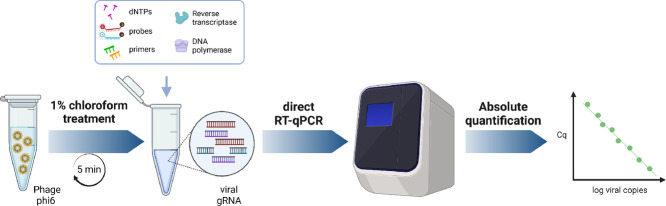

**Specifications table**

**Table:** 

Subject Area;	Biochemistry, Genetics and Molecular Biology
More specific subject area;	*Virology*
Method name;	*RT-qPCR for quantification of RNA phages*
Name and reference of original method;	*Louis Gendron, Daniel Verreault, Marc Veillette, Sylvain Moineau & Caroline Duchaine (2010)****Evaluation of Filters for the Sampling and Quantification of RNA Phage Aerosols****, Aerosol Science and Technology, 44:10, 893–901, DOI:*10.1080/02786826.2010.501351
Resource availability;	*Reagents* • UltraPlex® 1-Step ToughMix® Low ROX™ (4X), QuantaBio; product no. 95168–500 • Templates for RNA standards (Addgene: #101156 and #182535)

## Method details

### Description of protocol

The COVID-19 pandemic has called to attention the need for rapid yet sensitive and cost-effective methods for direct detection and quantification of airborne viruses. The most commonly used methods rely on isolating and purifying the viral RNA genome followed by reverse transcription (RT) and quantitative polymerase chain reaction (qPCR). However, the applicability of these methods has been hampered by the high demand for viral RNA purification reagents. A handful of direct (i.e. RNA extraction-free) RT-qPCR methods have been recently developed for SARS-CoV-2 virus detection [1,2]. As SARS-CoV-2 is a single-stranded positive-sense RNA (+ssRNA) enveloped virus [3], these direct detection approaches rely on removing the lipid envelope, either by heat-inactivation or by use of detergents, such as Igepal [1,2], prior to amplification.

**Fig. 1:**
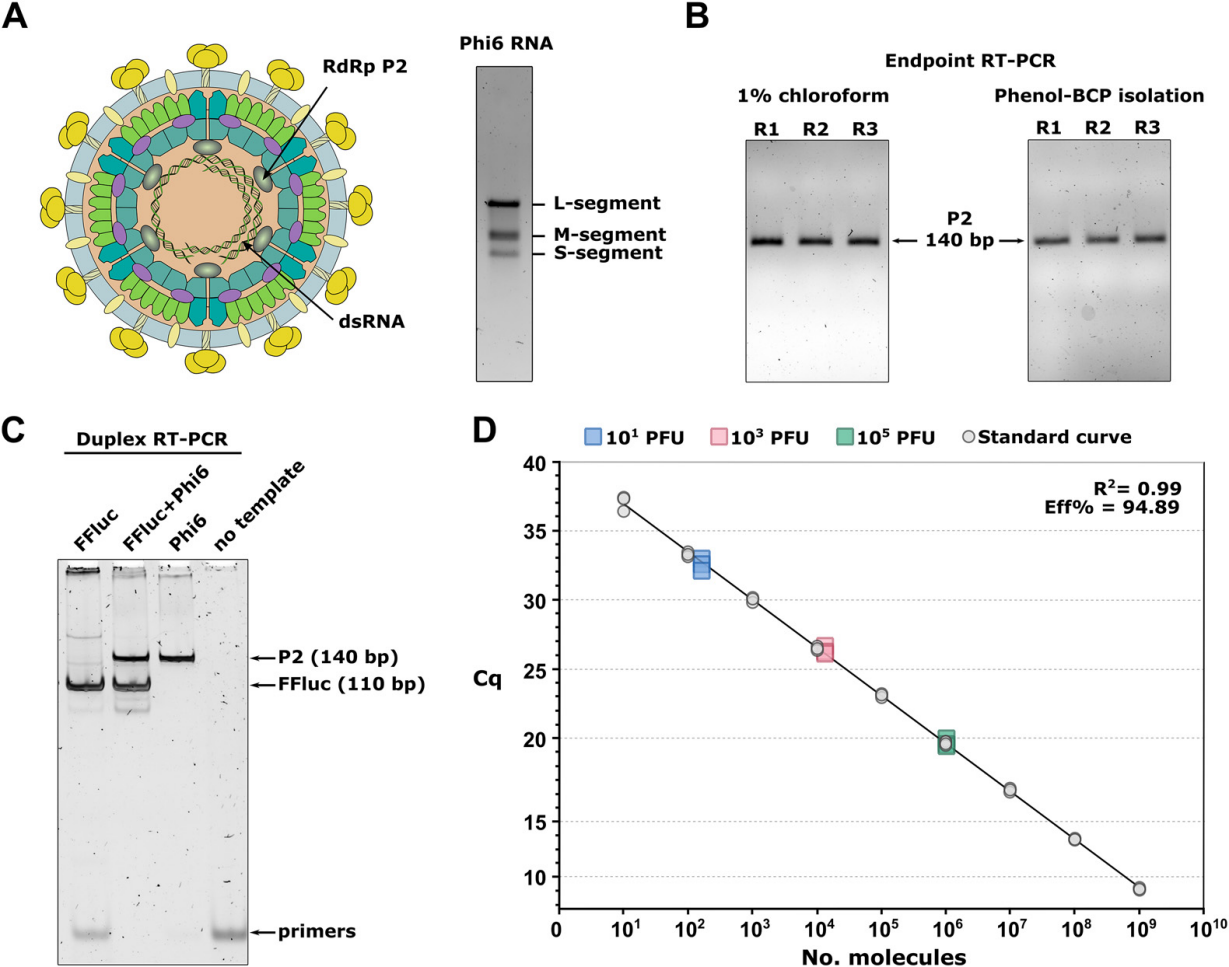
Optimization of the direct RT-qPCR method for phage phi6 detection. (**A**) Schematic representation of Pseudomonas phage phi6 (left panel; modified from ViralZone, Swiss Institute of Bioinformatics) and agarose gel electrophoresis of the genomic dsRNA segments (right panel). (**B**) Comparison of amplification efficiency of purified dsRNA (phenol-chloroform isolation) and chloroform treated viral samples by endpoint RT-PCR reaction. (**C**) Test of cross-reactivity of the target P2 and control Firefly Luciferase RNA (FFluc) primers in a multiplexed RT-PCR reaction. Each reaction consisted of both sets of primers, P2, and FFluc. (**D**) Standard curve for the absolute quantification of phage phi6 (round points). The 10^0^ dilution was not used for construction of calibration curve due to a non-linear response. The method was validated by quantifying three dilutions of 1x purified phage phi6 (rectangles). *n* = 3, primer effciency = eff%.

Since there are inherent risks in working with pathogenic viruses, bacteriophages are often utilized as safe-to-use alternative models [4], [5], [6]. Due to its structural resemblance to numerous pathogenic viruses, phage phi6 has become a popular analog for airborne transmission studies. Phage phi6 has a tripartite double-stranded (ds)RNA genome enclosed within a capsid (Fig. 1A). Stripping the lipid envelope weakens the capsid structure. During the RT step, the weakened capsid is prone to degrade due to the increased internal pressure caused by the elevated reaction temperature, thus exposing the genomic RNA and enabling direct RT-qPCR detection. Similarly, synthetically produced capsids (virus-like particles) of SARS-CoV-2 have been shown to be unstable and readily degrade as the temperature exceeds 34 °C [7]. The lipid envelope is frequently removed using various organic solvents [5], which often inhibit the enzymatic activity in downstream steps. Consequently, we tested the inhibitory effects of organic solvents (0.01% and 1% 1‑bromo-3-chloropropane, 0.01% and 1% chloroform) and a detergent (0.005% and 0.5% sodium deoxycholate) using end-point RT-PCR. The most efficient amplification and lowest inhibitory effect was observed when using 1% chloroform treatment, which was chosen as the standard treatment condition (Fig. 1B, Supplementary Fig. 1). It is worth noting that this approach is equally applicable for analyzing other enveloped viruses, although the inhibitory effects and efficacy of lipid envelope removal need to be tested separately for each virus. Importantly, quantification of phage phi6 is facilitated by the tightly regulated genome packaging mechanism, whereby the mature viral particle contains only one copy of each genome segment [8]. Therefore, the detection of any of the viral genes directly reflects the number of viral particles present in the sample. Moreover, we included a synthetic control template (Firefly Luciferase) to detect possible inhibitory effects of compounds present in the sample. In conclusion, this method provides a comprehensive protocol for the absolute quantification of phage phi6 and other enveloped RNA viruses by direct RT-qPCR using hydrolysis probes, as well as a protocol to enzymatically synthesize all necessary RNA standards.

*Required reagents and equipment*

• DNA template plasmids for preparation of RNA standard and control RNA, Addgene plasmids #101156 and #182535.
• Primers and hydrolysis qPCR probes (see Table 1), Metabion.

**Table 1:** List of used primers and probes.

Name	Sequence (5′->3′)	Amplicon size	Reference
Phi6_P2_qPCR-fwd	TGGGCGTCGATGAAAGATAC	140 bp	This study
Phi6_P2_qPCR-rev	TCGAGAGTGTCGCGTTTAAG		
Phi6_P2_probe-FAM^a^	**FAM**-ACGGTGCCTGTCCGATCTACTCTGA-**BHQ1**		
FFluc_qPCR-fwd	ATCGAGGTGAACATCACGTA	110 bp	This study
FFluc_qPCR-rev	TCACTGCATACGACGATTCTG		
FFluc –probe-TAMRA^a^	**TAMRA**-CATAGCTTCTGCCAACCGAACGGAC-**BHQ2**		

**Note:** Choice of fluorophore depends on the filters available in the Real-Time PCR system.

a Probes need to be aliquoted (5 µL) and stored at −20 °C, protected from light.
• P2 RNA standard and Firefly Luciferase RNA (prepared in this protocol – see Table 2).

**Table 2:** List of RNA standards used (*in vitro* transcribed = IVT RNA).

RNA	Plasmid template for IVT	Plasmid size	Addgene no.	Template linearization with	RNA size (ssRNA)^a^	MW of RNA^b^
P2	T7-Phi6-P2	5177 bp	#182535	*Eco*RI	1488 nt	478.16 kDa
FFluc	T7-CMVtrans-FFLuc-polyA	4331 bp	#101156	*Eco*RI	642 nt	205.58 kDa

a Complete sequence of RNA standard is available in the Supplementary Information section.

b MW was calculated using RNA Molecular Weight Calculator at https://www.aatbio.com/tools/calculate-RNA-molecular-weight-mw.
• FastDigest *Eco*RI, ThermoFisher Scientific, product no. FD0274. Note that the manufacturer does not provide a unit definition or concentration for this product.
• T7 polymerase (20 U/µL), ThermoFisher Scientific, product no. EP0111.
• DNase I (1 U/µL), RNase-free, ThermoFisher Scientific, product no. EN0521.
• RiboLock RNase inhibitor (40 U/µL), ThermoFisher Scientific, product no. EO0381.
• Agarose, Molecular Biology grade, Fisher BioReagents, product no. BP160–100, CAS: 9012–36–6.
• 1xTAE buffer (Tris-Acetate-EDTA buffer; 40 mM Tris, 20 mM sodium acetate, 1 mM EDTA).
• Razors or gel cutters.
• NTP Set (100 mM each), ThermoFisher Scientific, product no. R0481. Note that 25 mM dilution needs to be prepared.
• Phenol, saturated pH 4.3, Fisher Chemical, product no. BP1751I_400, CAS: 108–95–2.
• Ethanol (99.6%), grade Aa.
• E.Z.N.A.® Plasmid DNA Mini Kit I, (V-spin), Omega Bio-tek, product no. D6943–01.
• E.Z.N.A.® Gel Extraction Kit (V-spin), Omega Bio-tek, product no. D2500–01.
• Qubit® RNA BR Assay Kit, ThermoFisher Scientific, product no. Q10210.
• Chloroform, pure, Acros Organics, product no. 423550010, CAS: 67–66–3.
• UltraPlex® 1-Step ToughMix® Low ROX™ (4X), QuantaBio; product no. 95168–500.
• Adjustable pipettes (1–1000 µL)
• Tips with aerosol barrier (1–1000 µL), low retention.
• 1.5 mL tubes, low retention.
• Vortex.
• Benchtop microcentrifuge.
• Laminar cabinet for assembling qRT-PCR reactions (optional).
• Qubit® Fluorometer, ThermoFisher Scientific.
• QuantStudio™ 3 Real-Time PCR System, ThermoFisher Scientific (or another qPCR system).

*Procedure*

### Preparation of RNA standard and firefly luciferase control RNA

The single-stranded RNA standards used for generating the calibration curve can be prepared by *in vitro* transcription (IVT) using T7 polymerase. The template plasmids can be easily propagated in *Escherichia coli* and linearized with a single restriction enzyme (*Eco*RI). For RNA preparation, all buffers, reagents, and disposable material need to be RNase-free grade and good laboratory practices for work with RNA should be followed.

*Template preparation for in vitro RNA synthesis*

1. Propagate template vectors and isolate the plasmid. For plasmid isolation, we use E.Z.N.A.® Plasmid DNA Mini Kit I (Omega Bio-tek), but any other plasmid purification kit can be used.
2. Linearize the plasmid by *Eco*RI cleavage. An example for the reaction set-up is given in Table 3. Carry out the cleavage reaction for 1 h at 37 °C.

**Table 3:** Linearization of template plasmids.

Reagent	For 1 reaction	Final Conc.
10x FastDigest buffer Green	5 µL	1x
Plasmid (T7-Phi6-P2 or T7-CMVtrans-FFLuc-polyA)	5 µg	100 ng/µL
*Eco*RI	2 µL	–
Water, nuclease-free	Up to 50 µL	–
**Total reaction volume:**	**50** µL
3. Separate the linearized plasmid on a 0.8% agarose gel in 1xTAE buffer, extract the plasmid from gel and purify it using a gel extraction kit (recommended e.g. E.Z.N.A.® Gel Extraction Kit, Omega Bio-tek). Alternatively, the linearized plasmid can be purified directly, without agarose gel electrophoresis, using any PCR purification kit that is suitable for up to 10 kb DNA fragments. Store the purified DNA at −20 °C.

*In vitro RNA synthesis*

1. To synthesize single-stranded RNA, assemble the IVT reactions as described in Table 4.

**Table 4:** *In vitro* transcription reaction.

Reagent	For 1 reaction	Final Conc.
Linearized plasmid (T7-Phi6-P2 or T7-CMVtrans-FFLuc-polyA)	500 ng	10 ng/µL
5x Transcription buffer	10 µL	1x
25 mM NTPs (each)	4 µL	2 mM (each)
40 U/µL RiboLock RNase inhibitor	1.25 µL	1 U/µL
20 U/µL T7 RNA polymerase	1.5 µL	0.6 U/µL
Water, nuclease-free	Up to 50 µL	–
**Total reaction volume:**	**50** µL
2. Incubate the reactions at 37 °C for 2 h.
3. Remove the DNA template by adding 2 µL of DNase I, mix well and incubate at 37 °C for another 15 min. Proceed immediately with RNA purification.

*RNA purification*

1. Add 250 µL of nuclease-free water to the IVT reaction.
2. Add 300 µL of acidic phenol and 60 µL of chloroform.

Note: The purification reaction can be upscaled. The volume of added phenol should be equal to the total reaction volume in step 1, add chloroform at 1/5 of the phenol volume.

1. Vortex vigorously at full speed for 15 s.
2. Separate the phases by centrifugation at 10 000 *g* for 15 min at room temperature.
3. Collect the aqueous phase into a new tube and add 300 µL of isopropanol. Precipitate the RNA at RT for 30 min.
4. Pellet the RNA by centrifugation at 16 000 *g* for 30 min.
5. Wash the RNA by 1 mL of 80% ethanol. Vortex the sample vigorously.
6. Pellet the washed RNA by centrifugation at 16 000 *g* for 10 min.
7. Carefully remove the supernatant and air dry the RNA pellet.
8. Dissolve the pellet in nuclease-free water and determine the RNA concentration with Qubit BR RNA Assay kit. Optional: Check the quality of the purified RNA by agarose gel electrophoresis. Store the purified RNA at −80 °C.

The typical yield from this reaction is between 40 and 70 µg of purified RNA.

### Virus sample preparation before RT-qPCR

Note: Samples containing virus should be stored at −80 °C to preserve the RNA quality and obtain unbiased data.

1. Thaw the samples while mixing (done at room temperature, then moved immediately onto ice).
2. Transfer 100 µL of the sample into a new Eppendorf tube.
3. Add 1 µL of chloroform to each sample.
4. Vortex vigorously for 15 s. Next, incubate the samples for 5 min at room temperature.
5. Centrifuge the samples for 1 min at 10 000 *g*.
6. Place the samples on ice and proceed with quantification by RT-qPCR.

### Quantification of virus by RT-qPCR

The RT-qPCR enzyme mix used in this assay is designed for use with hybridization probes such as TaqMan®. The enzyme mix utilizes ROX passive reference and therefore, the instrument compatibility needs to be checked prior to designing the assay. The RT reaction can be carried out in a wide range of temperatures (42 °C to 60 °C), however 50 °C proved to be the most optimal (data not shown).

The assay design includes the Firefly Luciferase (FFluc) internal control template to detect possible inhibitory contaminants from the sample. The primer and probe designs were done using IDT PrimerQuest™ and primers and probes were checked for cross-reactivity with the IDT OligoAnalyzer™ tool. The primers and probes for the duplex reaction were designed according to the recommendations for multiplex qPCR design with minimal cross-reactivity between primers and probes (Fig. 1C). The selection of reporter dyes was limited by the availability of filters on the qPCR system. Consequently, we selected the reporter dyes with the smallest overlap in their emission spectra.

Furthermore, a wide range of primer/probe concentrations were tested in the RT-qPCR set-up to find the most optimal efficiency of the reaction. The final reaction mix constitutes the most efficient amplification (primer efficiency ∼95%) with the widest detection range (10^1^ – 10^9^ genome copies) (Fig. 1D).

1. Prepare the reaction mastermix as described in Table 5.

**Table 5:** Master mix for qRT-PCR.

Reagent	For 1 reaction	Final Conc.
4xUltraPlex® 1-Step ToughMix®	2.5 µL	1x
10 µM P2 fwd	0.3 µL	300 nM
10 µM P2 rev	0.3 µL	300 nM
10 µM FFluc fwd	0.3 µL	300 nM
10 µM FFluc rev	0.3 µL	300 nM
10 µM FFluc probe (TAMRA)	0.15 µL	150 nM
10 µM P2 probe (FAM)	0.15 µL	150 nM
FFluc standard (10^3^ molecules)	1 µL	10^3^ molecules
Water, nuclease-free	0 µL	–
**Template**	**5** µL	–
**Total reaction volume:**	**10** µL
2. Place the qPCR plate on ice and pipette 5 µL of the reaction mastermix into each well used of the qPCR plate.
3. Add 5 µL of template/well and seal the plate. As a negative control, add 5 µL of double-distilled H_2_O (ddH_2_O) instead of the template. All samples should be analyzed in technical triplicates.

Note: At the same time, construct the calibration curve using the P2 RNA standard (see section below).

4. Centrifuge the plate for 1 min at 1000 *g* to collect the reaction mix to the bottom of the wells.
5. Run the RT-qPCR reaction in a cycler using the program described in Table 6. Set the correct filters, the reaction volume to 10 µL, and the lid temperature to 105 °C.

**Table 6:** qRT-PCR cycling program.

		Temperature	Time	Ramp	No. of cycles	Notes
RT reaction and inactivation of RT enzyme	Step 1	50 °C	20 min	1.6 °C/s	–	–
	Step 2	95 °C	3 min	1.6 °C/s		
PCR reaction	Step 1	95 °C	10 s	1.6 °C/s	45 cycles	–
	Step 2	62 °C	1 min	2.5 °C/s		Data collection point

*Method validation*

To validate our method, we quantified three different concentrations of 1x purified virus diluted in Tris–HCl, pH 7.2 buffer (Fig. 1D, rectangles) using viral infectivity (PFU/reaction) as a measure for the virus amount. The analyzed samples contained 10^1^, 10^3^ and 10^5^ PFU/reaction. As expected, the amount of genome copies detected was approx. 10-fold higher than the infective counts. This difference can be attributed to the presence of non-infective virus particles. Despite the tightly regulated packaging mechanism of phage phi6, which ensures the addition of one copy of each genome fragment into the viral capsid [7], infectivity of the particle is also dependent on additional virus and host factors. Indeed, loss of infectivity of the mature virion may be ascribed to incomplete outer shell assembly, loss of the viral envelope, or loss of the spike protein, as well as to host factors, such as a reduced pili expression (entry path for phage phi6) by the bacterium [9].

### Establishing the calibration curves

1. Make a stock solution of the P2 RNA standard containing 10^10^ molecules/µL. For a calculation guide, please refer to the Supplementary Information.
2. Prepare a dilution series containing P2 RNA standard at concentrations from 10^0^ to 10^9^ molecules per 5 µL (as described in Table 7). Vortex thoroughly each dilution prior to making the next one.
3. Next, to ascertain the possible inhibitory effect of chloroform on the activity of the enzymes in the RT-qPCR mix, add chloroform to the dilutions at an amount equivalent to that used for the samples.
4. Vortex all dilutions and centrifuge the tubes at 10 000 *g* for 1 min.
5. Store the tubes on ice until assembling the reaction.
6. Prepare the RT-qPCR reactions as described above (section Quantification of virus by RT-qPCR, Table 5) and use the dilution series as a template. Each standard dilution should be analyzed in technical triplicates.
7. Create the calibration curve. A representative calibration curve is show in Fig. 1D, and a template for generating the calibration curve is provided in the Supplementary Information .

**Table 7:** Dilution series for generating the calibration curve.

Dilution (no. of molecules per 5 µL)	P2 (10^10^ molecules) RNA stock	ddH_2_O	Chloroform (pure)^a^
10^9^	2 µL 10^10^ P2 RNA stock	98 µL	0.9 µL
10^8^	10 µL of previous dilution	90 µL	0.9 µL
10^7^	10 µL of previous dilution	90 µL	0.9 µL
10^6^	10 µL of previous dilution	90 µL	0.9 µL
10^5^	10 µL of previous dilution	90 µL	0.9 µL
10^4^	10 µL of previous dilution	90 µL	0.9 µL
10^3^	10 µL of previous dilution	90 µL	0.9 µL
10^2^	10 µL of previous dilution	90 µL	0.9 µL
10^1^	10 µL of previous dilution	90 µL	0.9 µL
10^0^^b^	10 µL of previous dilution	90 µL	1 µL

a Chloroform is added after all dilutions have been prepared. Note that the total volume for the 10^0^ dilution is 100 µL and 90 µL for all previous dilutions.

b Optional. We observed that reactions with the 10^0^ dilution (i.e. 1 molecule/reaction) are outside of the linear detection range for this method using phage phi6.

### Data analysis

The data analysis was performed using the analysis module of QuantStudio™ Design and Analysis software, v1.5.1 (Thermo Fisher Scientific) using the standard curve generated as described above. Alternatively, the analysis can be performed using the formula below, where Cq is the quantitation cycle and Yintercept as well as the slope are derived from the calibration curve (Fig. 1D). An example of such a calculation is provided in the Supplementary Information.

$$Quantity\;per\;well=\frac{Cq-Yintercept}{slope}$$

## Conclusions

Here we describe a simple RNA-extraction free method for direct RT-qPCR quantification of phage phi6. Additionally, we provide a detailed protocol for in-house synthesis of RNA standards, making the method cost-effective and easy to implement. Similarly to other direct RT-qPCR methods, the limitations of this method are primarily dictated by the sample volume [2]. For example, some aerosol collection experiments may generate very large sample volumes, which introduces a dilution factor that could prevent quantification due to the limited amount of viral RNA genome copies that are transferred to the RT-qPCR reaction. To circumvent this limitation and ensure a reliable and quantitative outcome, the amount of chloroform added to the virus containing sample is kept to a minimum, whereas the maximal possible volume of the final processed sample is added to the RT-qPCR reaction.

## Declaration of Competing Interest

The authors declare that they have no known competing financial interests or personal relationships that could have appeared to influence the work reported in this paper.

## Appendix Supplementary materials

Image, application 1
