# Supplementary material for: An improved RT-qPCR method for direct quantification of enveloped RNA viruses
Source: MethodsX. 2022 May 23;9:101737. doi: 10.1016/j.mex.2022.101737 (PMC9162933; doi:10.1016/j.mex.2022.101737)
Supplement: Supplementary file 1 [file mmc1.pdf]

Method Article – Title Page

**Title**	*An improved RT-qPCR method for direct quantification of enveloped RNA viruses*
**Authors**	*Pavlina Gregorova, Minna-Maria Heinonen, Peter Sarin**
**Affiliations**	*RNAcious Laboratory, Molecular and Integrative Biosciences Research Programme, Faculty of Biological and Environmental Sciences, University of Helsinki, Finland*
**Corresponding Author's email address**	*peter.sarin@helsinki.fi*
**Keywords**	RT-qPCR, direct quantification, bacteriophage, Phi6, RNA virus, RNA-dependent RNA polymerase
**Direct Submission or Co-Submission**	*Direct Submission*

ABSTRACT

Reverse transcription quantitative PCR (RT-qPCR) has emerged as the gold standard for virus detection and quantification, being utilized in numerous diagnostic and research applications. However, the direct detection of viruses has so far posed a challenge as the viral genome is often encapsidated by a proteinaceous layer surrounded by a lipid envelope. This necessitates an additional and undesired RNA extraction step prior to RT-qPCR amplification. To circumvent this limitation, we have developed a direct RT-qPCR method for the detection of RNA viruses. In our method, we provide a proof-of-concept using phage phi6, a safe-to-use proxy for pathogenic enveloped RNA viruses that is commonly utilized in e.g. aerosolization studies. First, the phage phi6 envelope is removed by 1% chloroform treatment and the virus is then directly quantified by RT-qPCR. To identify false negative results, firefly luciferase is included as a synthetic external control. Thanks to the duplex format, our direct RT-qPCR method reduces the reagents needed and provides an easy to implement and broadly applicable, fast, and cost-effective tool for the quantitative analysis of enveloped RNA viruses.

- *One-step direct RT-qPCR quantification of phage phi6 virus without prior RNA isolation*
- *Reduced reaction volume for sustainable and cost-effective analysis*

SPECIFICATIONS TABLE

**Subject Area**	Biochemistry, Genetics and Molecular Biology
**More specific subject area**	*Virology*
**Method name**	*RT-qPCR for quantification of RNA phages*
**Name and reference of original method**	*Louis Gendron, Daniel Verreault, Marc Veillette, Sylvain Moineau & Caroline Duchaine (2010)* ***Evaluation of Filters for the Sampling and Quantification of RNA Phage Aerosols****, Aerosol Science and Technology, 44:10, 893-901, DOI: 10.1080/02786826.2010.501351*
**Resource availability**	*Reagents*<br>• UltraPlex® 1-Step ToughMix® Low ROX™ (4X), QuantaBio; product no. 95168-500<br>• DNA templates for RNA standards (Addgene: #101156 and #182535

Supplementary Figures

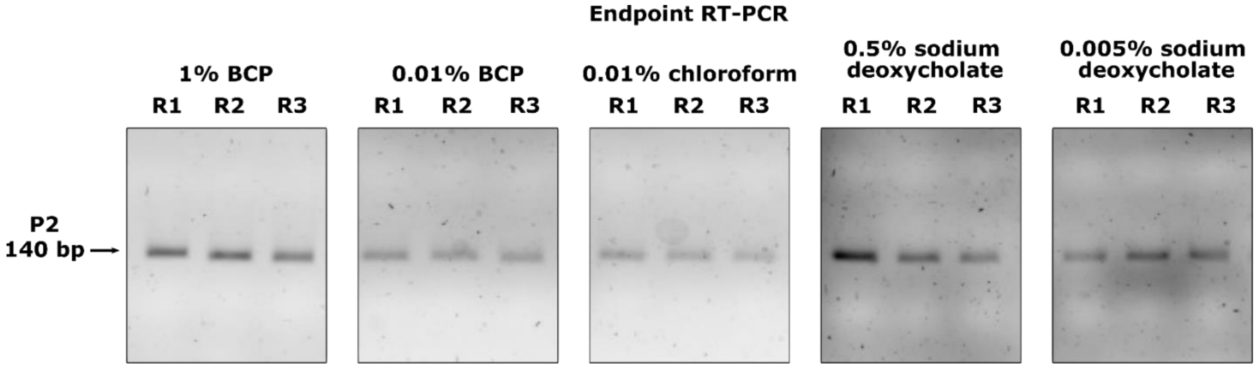

Suppl. Figure 1 – *Efficiency comparison of different sample treatment conditions following end-point PCR. BCP = 1-bromo-3-chloropropane. For 1% chloroform treatment and phenol-BCP isolated samples, see Figure 1B.*

Supplementary Material

Full sequences of RNA standards produced in the protocol

Forward primer Reverse primer Probe

```
>P2_RNA
GGGAGACCCAAGCUUCCAAUACUACCGGAAAAUUCGUGUCAAAGGAUCGUAUGGUCGCUGACUUCGAAUACGCAGUCACGGGCGGUGAGCAAGGCU
CGCUGUUCGCUGCUUCGAAGGAUGCCUCUCGUUUGAAGGAACAGUACGGGAUAGAUGUCCCGGACGGGUUUUUCUGCGAGCGGCGUCGUACCGCUA
UGGGUGGUCCGUUCGCGUUGAACGCUCCUAUCAUGGCCGUUGCGCAACCUGUGCGAAACAAAAUUUACUCCAAGUACGCUUACACCUUUCACCAUA
CUACUCGUCUUAAUAAGGAGGAAAAGGUGAAAGAGUGGUCGUUGUGCGUCGCUACUGACGUAUCCGACCACGACACGUUCUGGCCUGGAUGGCUGC
GGGAUCUCAUCUGUGAUGAACUGCUCAACAUGGGGUACGCUCCGUGGUGGGUUAAGUUGUUCGAGACCUCGCUCAAACUGCCCGUUUACGUGGGCG
CUCCUGCUCCUGAGCAGGGCCACACGUUGUUGGGUGAUCCGUCCAACCCUGAUCUCGAAGUUGGUCUCUCGUCCGGACAAGGGGCGACCGACCUCA
UGGGCACGUUGCUCAUGAGUAUCACCUACCUGGUGAUGCAACUUGAUCACACCGCUCCUCACCUCAACAGUCGAAUCAAGGACAUGCCAUCAGCAU
GCCGCUUUCUUGACUCGUAUUGGCAAGGACACGAGGAGAUCCGUCAGAUCUCAAAAUCUGAUGAUGCUAUGCUUGGCUGGACCAAAGGUCGUGCUU
UGGUUGGUGGUCAUCGUUUGUUCGAGAUGCUGAAAGAGGGUAAGGUUAACCCCUCACCUUACAUGAAGAUCUCCUACGAGCACGGUGGCGCCUUCC
UUGGUGACAUCCUGCUUUACGACUCGCGUCGUGAGCCUGGCUCUGCCAUCUUCGUUGGUAACAUCAACUCAAUGCUGAACAACCAGUUCAGCCCUG
AGUACGGUGUCCAAUCGGGCGUUCGCGACCGAUCUAAGCGCAAACGGCCGUUCCCCGGUCUUGCUUGGGCGUCGAUGAAAGAUACCUACGGUGCCU
GUCCGAUCUACUCUGAUGUGCUGGAGGCGAUCGAGCGUUGCUGGUGGAACGCGUUCGGUGAGUCGUACCGUGCGUAUCGUGAAGAUAUGCUUAAAC
GCGACACUCUCGAACUAUCACGCUACGUUGCGUCGAUGGCUCGUCAAGCCGGGCUGGCUGAACUCACUCCCAUUGAUUUGGAGGUGCUUGCUGACC
CGAACAAACUCCAGUAUAAGUGGACCGAGGCCGAUGUCUCGGCGAAUAUCCACGAGGUACUGAUGCAUGGCGUAUCGGUCGAAAAGACUGAGCGCU
UUCUCCGUUCUGUAAUGCCGAGGUAAUCAUGCCGAUUGUCGUAACUCAAGCGCAUAUUGAUCGUGUCGGCAUCGCCGCCGAUCUGCUCGAUGCGUC
UCCUGUGUCGCUUCAAGUUCUUGGUCGCCCUACCAGUAUUGGCGAAUU
```

```
>FFluc_RNA
GGGAGACCCAAGCUUUCAGAUCCGCUAGCGCUACCGGGGAUCCAGCCACCAUGGAAGACGCCAAAAACAUAAAGAAAGGCCCGGCGCCAUUCUAUC
CUCUAGAGGAUGGAACCGCUGGAGAGCAACUGCAUAAGGCUAUGAAGAGAUACGCCCUGGUUCCUGGAACAAUUGCUUUUACAGAUGCACAUAUCG
AGGUGAACAUCACGUACGCGGAAUACUUCGAAAUGUCCGUUCGGUUGGCAGAAGCUAUGAAACGAUAUGGGCUGAAUACAAAUCACAGAAUCGUCG
UAUGCAGUGAAAACUCUCUUCAAUUCUUUAUGCCGGUGUUGGGCGCGUUAUUUAUCGGAGUUGCAGUUGCGCCCGCGAACGACAUUUAUAAUGAAC
GUGAAUUGCUCAACAGUAUGAACAUUUCGCAGCCUACCGUAGUGUUUGUUUCCAAAAAGGGGUUGCAAAAAAUUUUGAACGUGCAAAAAAAAUUAC
CAAUAAUCCAGAAAAUUAUUAUCAUGGAUUCUAAAACGGAUUACCAGGGAUUUCAGUCGAUGUACACGUUCGUCACAUCUCAUCUACCUCCCGGUU
UUAAUGAAUACGAUUUUGUACCAGAGUCCUUUGAUCGUGACAAAACAAUUGCACUGAUAAUGAAUU
```
